# Transdermal Film Loaded with Garlic Oil-Acyclovir Nanoemulsion to Overcome Barriers for Its Use in Alleviating Cold Sore Conditions

**DOI:** 10.3390/pharmaceutics13050669

**Published:** 2021-05-07

**Authors:** Alshaimaa M. Almehmady, Sarah A. Ali

**Affiliations:** 1 Department of Pharmaceutics, Faculty of Pharmacy, King Abdulaziz University, Jeddah 21589, Saudi Arabia; 2 Department of Oral Diagnostic Sciences, Faculty of Dentistry, King Abdulaziz University, Jeddah 21589, Saudi Arabia; samali@kau.edu.sa

**Keywords:** acyclovir, bioavailability, ex vivo permeation, garlic oil, optimization, self-nanoemulsifying drug delivery system, herpes simplex virus

## Abstract

The exponentially mounting cases of herpes simplex virus infection or cold sores have become a serious global concern. Acyclovir (ACV) and garlic oil (GO)-loaded lipid nanocarrier could be a promising therapeutic approach in alleviating cold sores, as well as limiting the biopharmaceutical constraints associated with ACV absorption and therapeutic efficacy. Therefore, the objective of the current research study was to formulate an ACV-GO self-nanoemulsifying drug delivery system (ACV-GO-SNEDDS) as transdermal films. The prepared SNEDDS was optimized using an experimental mixture design. The optimized ACV-GO SNEDDS was loaded in transdermal film and was evaluated for ex vivo skin permeation and in vivo pharmacokinetic prospects. An optimized ACV-GO SNEDDs formulation constituted of 10.4% (*w/w*) of GO, 64.8% (*w/w*) of surfactant mixture (Tween 20^®^-Span 20^®^); 24.8%(*w/w*) of co-surfactant (Propylene glycol^®^), and 200mg of ACV, respectively, were prepared and characterized for particle size (Y). The observed globule size of the optimized ACV-GO SNEDDS is 170 ± 13.45 nm. The results of stability studies indicated that the stability index of optimized ACV-GO-SNEDDS was more than 92 ± 3%. This optimized ACV-GO SNEDDS was loaded in hydroxypropyl cellulose transdermal film. The outcome of the ex vivo skin permeation study demonstrated a 2.3-fold augmented permeation of ACV from the optimized ACV-GO SNEDDS HPC transdermal film in comparison to the raw ACV transdermal film. There was a 3-fold increase in the relative bioavailability of the optimized ACV-GO SNEDDS transdermal film compared to the raw ACV-HPC film. The study findings confirmed that the ACV-GO SNEDDS transdermal film exhibited excellent potential to enhance the bioavailability of ACV.

## 1. Introduction

A massive proportion of the global population suffers from cold sores. Cold sores primarily affect the oral and perioral region, prompted by herpes simplex virus type-1 (HSV-1), and sometimes by herpes simplex virus type-2 (HSV-2) also, although this primarily affects the genital area [1,2]. Demographic updates provided by the World Health Organization (WHO) revealed that at present around 3.7 billion people are infected with HSV-1, which represents 66.6% of the world’s population within the age group of 0 to 49 [1,2]. Cold sores are usually characterized by pain, discomfort as prodromal signs, and the appearance of papules or vesicles that burst and form scabs, which falls off after some time, and lesions persist healing in due course of time [3,4,5]. To date, there is no current therapeutic regime for treating recurrent cold sores. For symptomatic relief, there are topical applications constituted of natural remedies, photodynamic therapy, heat or laser treatment, and antiviral medications [3,4,5]. HSV-1 has staggering health effects, and therefore there is an urgent necessity to develop advanced treatment modalities.

Acyclovir is one of the most efficacious and frequently used drugs for treating HSV infection [6,7,8,9]. However, some biopharmaceutical challenges associated with this drug affect its therapeutic efficacy [8,9,10,11]. Acyclovir (ACV) has high aqueous solubility (1.2 mg/mL), although its low permeability is the major constraint that led to its poor absorption and suboptimal therapeutic effects [10,12]. Acyclovir falls under Biopharmaceutical Classification System (BCS) class III, and the limiting step in its absorption is its membrane penetrability [10,12]. Various researchers worldwide have tried different formulation strategies to enhance its absorption such as conjugation with cyclodextrin [11], niosomes [6], and chemical modification [12]. With the chemical modification and cyclodextrin approaches, there was no significant improvement in permeation of ACV, but with niosomal dispersion, the relative bioavailability of ACV was increased by twofold in comparison to the free drug solution [6]. However, the drawbacks of ACV niosomes were its bigger mean vesicle size of 0.95 μm and percent drug entrapment of 11% [6].

ACV commercial formulations are widely available via different routes of administration. The oral ACV tablets are of very high concentration (200 mg) and are supposed to be taken five times a day to achieve the desired ACV concentration in plasma. Therefore, patient compliance and the serious adverse effects related with such high doses of ACV are the biggest constraints with ACV oral delivery [13]. Nonetheless, various nanoformulation approaches such as lipid based nanoformulations and polymeric nanoparticles of ACV have been tried to enhance the oral bioavailability of ACV. Consequently with bolus IV injection of ACV (100 mg/kg), which is given in serious cases of HSV infection, there is huge possibility of deposition of ACV crystals in the kidney that could lead to intratubular renal damage and renal failure [14,15]. To address this inherent issue of poor acyclovir bioavailability and dose related toxicity, IV stealthy nanoparticles using polylactic coglycolic acid (PLGA) were developed and the results were quite encouraging [16]. In light of such concerns with oral and IV administration of ACV, the topical and transdermal route has garner significant attention as this route seems to be the safest and most promising for the ACV delivery. However, ACV ointment has been reported to have only moderate efficacy owing to difficulties in penetrating the epidermal layer of the skin [17]. Here, novel nanoformulation approaches can contribute significantly in enhancing the permeation across epidermal skin layers and thus efficacy of transdermal ACV delivery.

Among different commercially available topical products, Zovirax^®^ Cream (Zovirax^®^; Glaxo Wellcome Operations, Barnard Castle, England) is the most frequently prescribed antiviral cream for treating symptoms of cold sores, which is composed of 5% ACV as an active ingredient. Although Zovirax^®^ does relieve pain and discomfort, its use is nevertheless restricted because of associated adverse side effects [18,19]. Besides hypersensitivity reactions to excessive exposure, difficulty in urination, swelling of feet, and shortness of breath are the severe concerns that mitigates patient use of Zovirax^®^ [18,19]. Therefore, a novel formulation approach could provide a better alternative in this scenario. Encapsulation of ACV in oil globules in the nano range might enhance its permeation and thus absorption. That is why in the present work, a self-nanoemulsifying drug delivery system (SNEDDS) loaded with ACV was investigated to enhance its permeation and thus its therapeutic effect.

SNEDDS are isotropic combinations of surfactants, solvents, and oils that instinctively emulsify in aqueous phase to produce smooth oil-in-water (*o*/*w*) nanoemulsion having a 20 to 200 nm globule size range [20,21]. SNEDDS have many benefits to offer for enhancing a transdermal drug delivery system (TDDS) of hydrophilic drugs such as ACV [22]. Various research studies supported the efficacy of SNEDDS for improving permeation of significant drug candidates such as meloxicam and curcumin via transdermal route [23,24]. SNEDDS of ACV via TDDS would lead to sustained and steady release of the drug that is needed for alleviating cold sores. Moreover, TDDS of ACV SNEDDS would also lead to enhanced patient compliance and avoidance of hepatic first-pass metabolism [25].

The clinical benefits of ACV are very limited [6,11,12]; thus, to enhance its therapeutic efficacy, garlic oil (GO) was used for ACV encapsulation. Pre-clinical investigation results confirm the antiviral efficacy of GO against HSV infection [26,27,28,29,30]. Research studies have demonstrated that garlic’s components, particularly thiosulfinates, have tremendous antiviral potential for HSV, and they apply their antiviral attributes via interacting with the surface charge of viral cell molecules and successively inhibiting viral access to host cells [29,30]. Formulating SNEDDS of GO as the oily constituent of the ACV nanoemulsion is expected to enhance antiviral activity substantially and thus alleviation of the cold sore more efficaciously, in contrast to using ACV alone encapsulated in a non-medicated oily constituent. Furthermore, combination of GO and ACV if proven effective, could minimize ACV dose and dose related its toxic effects.

Hence, the purpose of the present study was to formulate ACV-GO encapsulated SNEDDS. The prepared SNEDDS was optimized using an experimental mixture design. The effect of formulation variables on response parameters was studied using response surface methodology (RSM) to optimize a finalized formula to meet all desired attributes. The optimized ACV-GO SNEDDS was loaded in transdermal film and was assessed for in vivo pharmacokinetic fate and ex vivo skin pervasion. Such a combination of ACV-GO SNEDDS via transdermal route was anticipated to be a promising therapeutic approach in terms of a combination of benefits of two potential antiviral agents, enhancing permeation via SNEDDS, and prolonging and sustaining multifold enhanced antiviral activity to relieve cold sore conditions.

## 2. Materials and Methods

Acyclovir was purchased from Acros Organics, Morris Plains, NJ, USA. Tea tree oil, garlic oil, peppermint oil, sandalwood oil, anise oil, oregano oil, chamomile oil, lemon palm oil, thyme oil, and eucalyptus oil were procured from Sigma Aldrich, St. Louis, MO, USA. Oleic acid, PEG 200^®^, PEG 400^®^, Labrasol^®^ Propylene Glycol^®^ and Transcutol^®,^ Tween80^®^, span80^®^, tween60^®^, span60^®^, tween20^®^, span20^®^, hydroxyl propyl cellulose, and silicone were purchased from Gattefosse (Saint Priest Cedex, France). The chemicals used in the study consisted of analytical grade.

## 3. Estimation of Acyclovir (ACV) Solubility in Various SNEDDS Components

### 3.1. Screening of Oils

Different oils reported to have potential antiviral activity and those held to be efficacious against cold sores were screened for solubility of ACV. These oils included tea tree oil, garlic oil, peppermint oil, sandalwood oil, anise oil, oregano oil, chamomile oil, lemon palm oil, thyme oil, and eucalyptus oil. For assessing the saturation solubility of ACV in dissimilar oils, a surplus quantity of drug was included in 3 mL of oils, and the mixtures were constantly agitated to achieve equilibrium for 72 h at 25 °C in a mechanical shaker. Subsequently, these oil and ACV mixtures were subjected to centrifugation (Model R 23 Remi Instruments, Ltd., Mumbai, India) at 5000 rpm for 30 min, followed by separation of the supernatant, which was then dissolved in methanol to measure its solubility by UV spectrophotometer at 252 nm. The procedure was performed thrice, and the data was reported as ±SD. The oil in which ACV exhibited maximum solubility was selected as the oil phase for preparing nanoemulsion [31].

### 3.2. Screening of Surfactants and Co-Surfactants

Various surfactant mixtures whose Hidrophilic lipophilic balance (HLB) values correspond to those required by selected oil (HLB = 14) were tested for determining ACV solubility. The tested surfactants are Tween80^®^:Span80^®^:(9:1), Tween60^®^:Span60^®^ (9.4:0.6), and Tween 20^®^:Span20^®^ (6.67:3.33).

The percentage of each surfactant in surfactant mixture was calculated according to the following equation:

% of Surfactant with higher HLB = HLB req − HLB low/HLB high − HLB low × 100

Then the percentage of surfactant with lower HLB calculated according to (100% of surfactant with higher HLB). The co-surfactants tested for ACV solubility include PEG 200^®^, PEG 400^®^, Labrasol^®^ Propylene Glycol^®^, and Transcutol^®^. For conducting surfactant screening, the oil and surfactant mixtures were gently heated at 50 °C. Subsequently, a 500 mg portion of every mixture was correctly measured and carefully watered down with 9.5 mL double-distilled water to produce 10 mL of a smooth emulsion. The ease of emulsion formation was observed thoroughly. The resulting emulsions were diligently observed for optical clarity, homogeneity, and turbidity [31,32].

### 3.3. Pseudo Ternary-Phase Diagram for Preparing ACV SNEDDS

A pseudo ternary-phase diagram illustration of ACV was created on the basis of ACV solubility in the designated SNEDDS component arrangements to identify their suitable concentration for formulating nanoemulsions. Pseudo ternary-phase illustrations were constructed on the basis of observation of aqueous titration experiment, which was conducted by drop-wise accumulation of double-distilled water to the mixture of the selected oil, selected surfactants, and co-surfactant system. The concentration at which visually clear dispersions with a slight bluish tint was obtained was determined to fall in the nanoemulsion section of the phase diagram [21,31,32].

Briefly, an accurately weighed 2 g mixture of components of nanoemulsion was primed by adding variable quantities of the surfactant, co-surfactant, and oil. This component mixture was thoroughly mixed with a vortex mixer. The nanoemulsion formed upon addition 20 mL of distilled water to 100 mg of this component mixture was observed carefully for visual clarity and transparency [21].

### 3.4. Optimization of ACV-GO SNEDDS

The main purpose of applying the statistical design for optimization of ACV-GO SNEDDS is to understand more profoundly the influence of independent formulation variables on dependent response parameters to obtain the formulation with the best attributes within the definite concentration limits of SNEDDS components. The three independent variables were constituted of GO %*w*/*w* (X1), surfactant mixture %*w*/*w* (X2), and co-surfactant %*w*/*w* (X3). The measured response was particle size. In accordance with the overall run provided by the experimental design matrix by Design-Expert^®^ software (version 7; Stat-Ease, Inc., Minneapolis, MN, USA), 14 ACV-GO formulations were prepared and characterized (200 mg of ACV in each formulation), as shown in Table 1.

The concentration range of SNEDDS components or formulation variables were selected on the basis of the results of screening studies of SNEDDS components as described in the previous sections. According to which GO (X1) concentration varied from 8–20%*w*/*w*, surfactant mixture (X2) varied between 60–72%*w*/*w*, and co-surfactant (X3) fell within 20–32%*w*/*w*. The globule size (Y1) was used as the response parameter (dependent variable). The optimum formulation of this study was selected on the basis of smallest possible globule size.

### 3.5. Characterization of ACV-GO SNEDDS

#### 3.5.1. Emulsification Ability

The efficacy of the formulated ACV-GO SNEDDs was examined on the basis of their ability to emulsify spontaneously and form transparent dispersions. The systems were evaluated by observing the ability of prepared nanoemulsions to disappear from their crude emulsion state and form transparent and clear dispersions of nano-sized *o*/*w* globules [32,33].

#### 3.5.2. Determination of Globule Size of the ACV-GO SNEDDs

The particle size of ACV-GO SNEDDS was measured by dynamic light scattering (DLS) via Zetasizer (Zetatrac, Microtrac, and Montgomeryville, PA, USA). About 250 µL of the formulated dispersion was diluted with 750 µL of distilled water to obtain 1 mL aliquot for analysis of mean globule size of the prepared formulation. The globule size was measured on the basis of Brownian motion utilized by DLS, in which the fluctuations in light intensity scattered by nanoparticles undergoing consistent oscillation was analyzed.

#### 3.5.3. Evaluation of Stability of the Optimized ACV-GO SNEDDS

The optimized ACV-GO-SNEDDS formulation was characterized for thermodynamic stability studies by subjecting it to various temperature changes. The optimized ACV-GO-SNEDDS formulation passed through three consecutive freeze–thaw cycles (freezing at −25 °C for approximately 12 h and thawing at +25 °C for 12 h). After completing consecutive stress cycles, the optimized ACV-GO-SNEDDS formulation was analyzed for globule size. The stability index of optimized ACV-GO SNEDDS was computed by comparing the globule size with that of the initially measured globule size using the following equation:

Stability index = ([Initial size − Change in size]/Initial size) × 100

#### 3.5.4. Preparation of ACV-GO SNEDDs Transdermal Films

The optimized ACV-GO-SNEDDS were loaded in transdermal film. Accurately measured 15 mL of 1% hydroxyl propyl cellulose (HPC) was used as a film-forming polymeric solution, which was mixed with 5 mL of ACV-GO SNEDDs to prepare a solution with ACV concentration of 50 mg/mL. In a dish area equivalent to 50 mg drug/cm^2^, propylene glycol (1%*w*/*v*) was added to the solution as a plasticizer. The forming solution was exposed to mild mixing and after that left for 24 h at (8 °C) to obtain a fine solution. The primed gel was then decanted into 5-cm length silicon-coated Petri dishes for calmer exclusion of films. Petri dishes were then placed in an oven at 40 °C until the water entirely disappeared. The layers were then enclosed in an assistance membrane and cut into appropriate sizes. The quantified weight of ACV (similar as that in ACV-GO SNEDDs) was dispersed in an aqueous polymer solution for preparing raw ACV transdermal film (formulation F2). The formulation F3 was prepared by using oleic acid instead of GO, similar to that for ACV SNEDDS.

### 3.6. Ex Vivo Skin Permeation Study of ACV-GO SNEDDS Transdermal Films

The evaluation of diffusion and permeation efficiency of ACV from the prepared transdermal films (F1, F2, F3, and commercially available 5% ACV cream) was performed using the automated Franz diffusion cell technique (Hanson research, MicroettePlus, Chatsworth, CA, USA) with a 1.76 cm^2^ dissemination zone and a receptor compartment of 7 mL in volume. A fresh skin sample with complete thickness area of 2 cm by 2 cm unrestricted by any dermal fat was incised from the abdominal sections of clean-shaven male Wistar rats of 200–250 g weight. The collected skin was scanned with a magnifier to settle skin integrity. The prepared skin was tightly mounted between the donor and receptor compartments of the diffusion cell with the dermal side in direct contact with the receptor medium. An accurately weighed ACV formulation equivalent to 50 mg was placed in the donor compartment. Phosphate buffer saline (pH 7.4) was used as a diffusion medium in the receptor chamber, in which the temperature was kept at 32 ± 0.5 °C, and the stirring rate was 400 rpm. Aliquots withdrawn by the auto sampler were analyzed by reverse-phase high-performance liquid chromatography (RP-HPLC), according to the previously reported validation method. In which the chromatographic separation was performed on a Luna (2) RP-18 (250 by 4.6 mm, 5 μm packing) reverse phase analytical column (Chromatec) at 40 °C. The mobile phase was composed of methanol, water, and acetic acid at a ratio of 30:69.5:0.5 (pH 2.8). The mobile phase was pumped isocratically at a flow rate of 1.0 mL per minute.

### 3.7. Pharmacokinetic Evaluation of the Optimized ACV-GO SNEDDs Transdermal Film

#### 3.7.1. Animals

For evaluating pharmacokinetic fate of optimized ACV-GO-SNEDDS formulation, male Wistar rats of 200–250g body weight were used. These rats were administered a standard diet under meticulously maintained environments of humidity (30–70% relative humidity) and temperature (24 ± 2 °C). Ethical approval was provided by the local Institutional Review Board for Preclinical & Clinical Research within the constraints of proper care and cautious use of animals as per the Declaration of Helsinki and the Guiding Principle in Care and Use of Animals (DHEW publication NIH 80-23) and Principles of Laboratory Animal Care (NIH publication #85-23, revised 1985). The protocols were approved by the Institutional Review Board for Animal Research/Studies Animals, and rats were procured from the animal house facility of the Clinical Laboratory center, Beni-Suef, Egypt. (Approval No. 23-2-21)

#### 3.7.2. In Vivo Investigation of the Optimized ACV-GO SNEDDs Transdermal Film

For conducting in vivo study, the animals were divided into three groups. All these groups were given prepared formulations corresponding to dose per 10 mg/kg body weight via transdermal route with application area measuring 1.0 cm^2^ in area. Group 1 animals were given raw ACV-HPC film; Group 2 animals were given optimized HPC ACV-GO SNEDDs film; and Group 3 animals were treated with commercially available ACV 5% cream. The medicated transdermal ACV films (F1 and F2) were enclosed by plain adhesive patches. Pharmacokinetic calculations were computed on the basis of plasma ACV concentrations. The plasma drug concentration was analyzed by HPLC, as mentioned in the previous section. Kinetica (Version 4, Thermo Electron Corporation, Waltham, MA, USA) was used to compute the following pharmacokinetic parameters: maximum plasma concentration (C_max_), time point of maximum plasma concentration (t_max_), area under the plasma concentration–time curve (AUC), and elimination rate constant (k_e_).

### 3.8. Statistical Analysis

The results of the present investigation were expressed as the mean ± the standard deviation. The comparisons between the results of different study groups were estimated statistically using the paired *t*-test.

## 4. Results and Discussion

### 4.1. Estimation of Acyclovir (ACV) Solubility in Various SNEDDS Components

**Figure 1:**
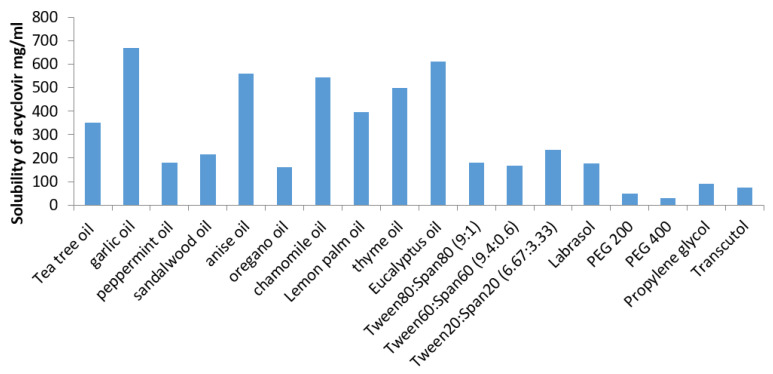
Solubility of acyclovir in different oils, surfactants, and co-surfactants. (Data are expressed as mean ± SD (*n* = 3)).

The self-emulsifying preparation consisting of oil, surfactant, co-surfactant, and drug should be visually clear and form a monophasic liquid when introduced into an aqueous system. Such a system should have good solubilizing ability to dissolve the drug and represent a drug solution. The solubility of ACV in different vehicles is shown in Figure 1. Amongst the different oils screened, ACV exhibited maximum solubility in GO (670 mg/mL). Therefore, GO was selected for the oil phase to prepare ACV SNEDDS. Amongst the different surfactant mixtures with HLB equal to 14 (corresponding to the Required HLB value of GO), ACV has shown the highest solubility in Tween20^®^:Span20^®^ (6.67:3.33). Therefore, based on these solubility results, GO and Tween20^®^:Span20^®^ (6.67:3.33) imparted the most suitable oil phase and surfactant mixture, respectively, to dissolve acyclovir.

For preparing ACV-SNEDDS, oil phase (GO) was selected on the basis of ACV’s highest solubility in it whereas surfactant and co-surfactant system was selected on the basis of their emulsification ability which means their ability to form transparent dispersions when their aqueous solutions were added to oil phase as per the procedure given under methodology (screening of surfactants). Although ACV exhibited maximum solubility in Labrasol but the dispersion obtained on its addition were turbid in appearance, therefore it was not selected as surfactant for formulating ACV SNEDDS.

The results of screening studies demonstrated that Tween20^®^:Span20^®^ and Propylene glycol^®^ surfactant and co-surfactant system exhibited the highest emulsification efficiency prerequisite for the formation of homogenous emulsion. The HLB values of the used surfactants and oils were analogous (HLB = 14); thus, the difference observed in the emulsifying ability with different surfactant mixtures can possibly be attributed to their dissimilar structure, chain length, and composition ratios [21,32].

### 4.2. Pseudo Ternary-Phase Diagram

**Figure 2:**
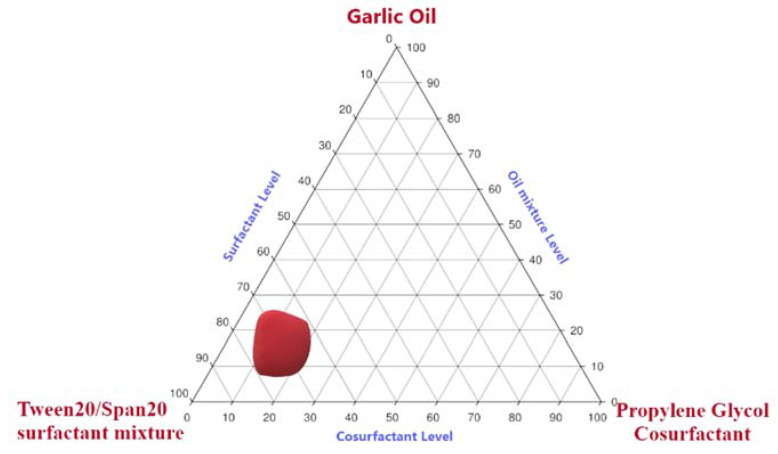
Pseudo ternary-phase diagram of selected components dispersed in water at 25 °C. Highlighted area represents the nanoemulsion region.

On the basis of screening the results of SNEDDS components, different phase diagram formulations were constructed by changing the proportion ranges of selected oils, surfactants, and co-surfactants. The phase diagram is represented in Figure 2. The dark-shaded region specifies the region of nanoemulsification. The wider this region, the better its self-nanoemulsifying ability [33]. In the present work, it can be seen that selected oils, surfactants, and co-surfactants caused wider nanoemulsification regions, indicating noteworthy self-nanoemulsification ability of the prepared systems. GO reportedly has an HLB value of 14, which is comparable to the HLB value of surfactant mixture, and as we know that when the HLB value of the surfactant system is close to that of the oil, the nanoemulsion gains the most stable status with the smallest globule size. On this basis, Tween 20^®^-Span 20^®^ (HLB-14) was added to GO to prepare a nanoemulsion that would form clear dispersion upon dilution. Addition of co-surfactant propylene glycol would have caused more water penetration and self-dispersibility of nanoemulsion formulations by possibly increasing the interfacial fluidity of surfactant boundaries in the micelles. The entrapment of GO in the high HLB surfactant and co-surfactant was anticipated to contribute to improving the emulsification process upon dilution with aqueous medium [21,31,32,33].

From the pseudo ternary-phase diagram, the concentration range selected for GO corresponds to 8 to 25%, Tween20^®^-Span20^®^ surfactant mixture corresponds to 60–80%, and Propylene glycol^®^ co-surfactant corresponds to 20–40% based on the nanoemulsion region obtained (Figure 2).

### 4.3. Optimization of ACV-GO SNEDDS

The SNEDDS components concentration ranges for optimization study were selected on the basis of the obtained pseudo ternary diagram with the maximum nanoemulsification region. Therefore, to optimize the particular concentrations of oil, surfactant, and co-surfactant that would lead to the smallest globule-sized ACV-GO SNEDDS, an optimal mixture experimental design was applied in this study. The three independent variables were GO %*w*/*w* (X1), surfactant mixture (Tween 20^®^/Span 20^®^) %*w*/*w* (X2), and Propylene glycol^®^ co-surfactant %*w*/*w* (X3). The measured response was globule size (Y1).

The responses of the prepared formulations are presented in Table 1. The influence of formulation variables on response parameter can be explicitly inferred with the aid of a polynomial equation generated by statistical analysis through Design-Expert^®^ software [32,34]. The larger coefficient value represents more pronounced influence on the response.

### 4.4. Effect of Formulation Variables on Particle Size

From the results obtained for impact of independent variables on globule size, it can be seen that all of the formulation variables have significant and direct effects on globule size in the order of: %*w*/*w* of co-surfactant (X3) > %*w*/*w* of garlic oil (X1) > %*w*/*w* of surfactant mixture (X2). Increases in the concentration of GO, surfactant mixture, and propylene glycol lead to significant increase in the globule size of ACV-GO SNEDDS. What is more, it can be seen very clearly in the polynomial equation generated by software that the combination effect of formulation variables is very profound on globule size. The largest globule size was found for F1 (250 nm), and the smallest globule size was observed for F14 (170 nm).

Globule size = +239.55A + 215.05B+249.05C − 104.82AB − 256.45AC − 127.45BC + 910.91A ² BC − 1411.09AB²C − 262.41ABC²

The results of ANOVA as summarized in Table 2 confirmed that the model was significant, and a statistically significant correlation was found between globule size and each of the independent formulation variables.

**Figure 3:**
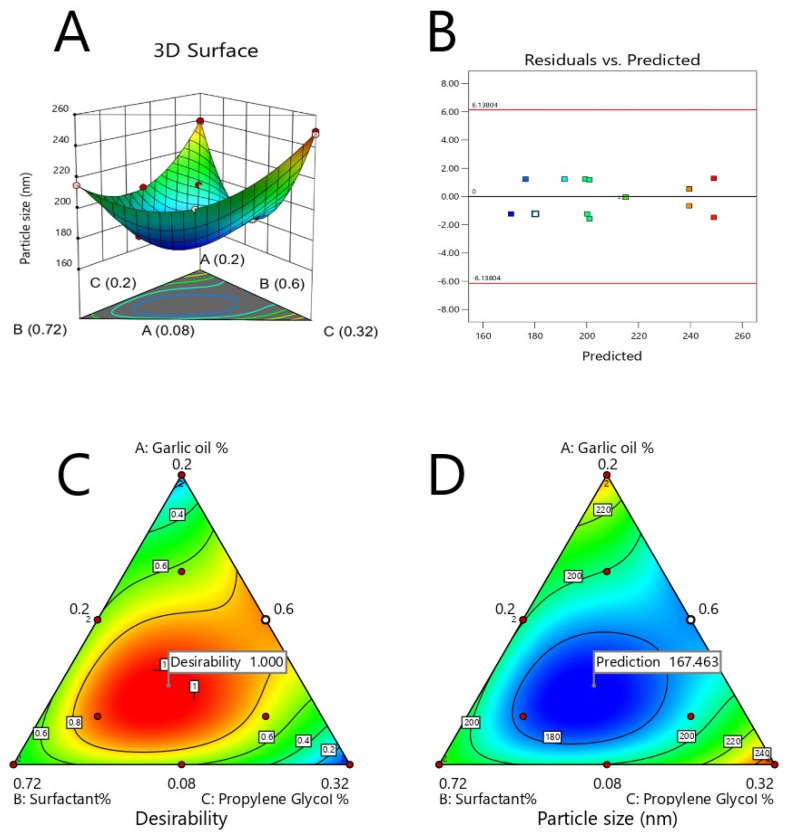
Graphical representation of the effects of independent variables on particle size of ACV-GO SNEDDS: (**A**) 3D-response surface plots, (**B**) Residual versus predicted plot, (**C**) contour plot showing effect of ACV-GO SNEDDS content on the desirability (**D**) contour plot showing effect of ACV-GO SNEDDS content on the response parameter.

**Figure 4:**
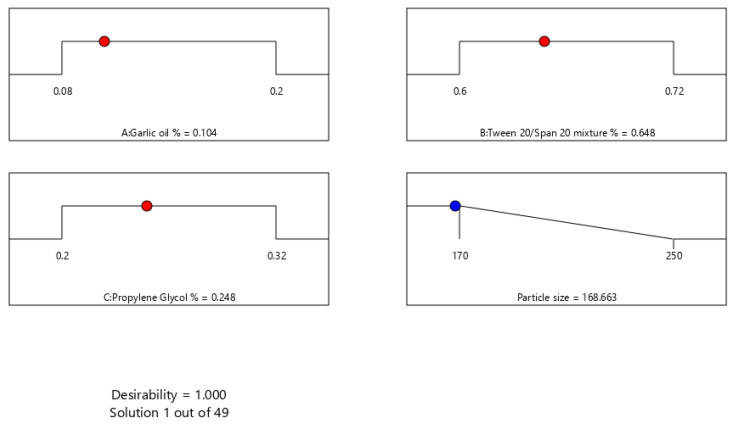
Desirability values of the numerical optimization process for ACV-GO SNEDDS through statistical design optimization.

The major purpose of optimization of ACV-GO-SNEDDS formulations is to find the synchronized concentration points of the different formulation variables at which robust formulations with excellent characteristics can be designed. The response parameter—that is, globule size—has to be minimized in the present optimization process. A SNEDDS formulation satisfying this criterion was formulated and assessed. The optimization evaluation analysis of ACV-GO SNEDDS indicated good agreement between the model prediction and experimental observation, confirming the validity of the model. Figure 3 represents an acceptable region that has met the requirement. An optimized formula was generated by a numerical optimization process following the desirability approach for ACV-GO SNEDDS formulation. Figure 4 represents the desirability values of the numerical optimization process. The outcomes of predicted and observed mean response for globule size of the optimized ACV-GO SNEDDS formulation with maximum desirability are 168.33 nm and 170 nm, respectively.

An optimized ACV-GO SNEDDs formulation that was constituted of 10.4% (*w/w*) of garlic oil (X1), 64.8% (*w/w*) of surfactant mixture (Tween 20^®^-Span 20^®^) (X2), 24.8% (*w/w*) of Propylene glycol^®^ co-surfactant (X3), and 200mg of ACV, respectively, were prepared and characterized for particle size (Y). The observed globule size of optimized ACV-GO SNEDDS is 170 ± 13.45 nm.

### 4.5. Emulsification Ability of ACV-GO SNEDDS

The emulsification ability of prepared ACV-GO SNEDDS is a significant indicator of the self-emulsification efficiency of formulations that dictates the capability of nanoemulsion to disperse completely and quickly when subjected to dilution under mild agitation [32,35].

All the prepared formulations rapidly emulsified and formed visually clear preparations on infinite dilutions. The rapid emulsification ability of prepared dispersions is attributed to the compositions of SNEDDS with lower oil content and higher surfactant and co-surfactant content, which resulted in lower viscosity and higher emulsification ability of the system [35].

### 4.6. Evaluation of Stability of Optimized ACV-GO SNEDDS

The optimized ACV-GO SNEDDS was tested for its thermodynamic stability, and there was no sign of precipitation, phase separation, coalescence, or creaming. The optimized SNEDDS formulations passed the thermodynamic stability test. The results of the thermodynamic stability studies demonstrated that the stability index of optimized ACV-GO-SNEDDS was more than 92 ± 3%.

This optimized ACV-GO SNEDDS was loaded in HPC transdermal film (F1). The F1 (optimized ACV-GO SNEDDS) was loaded in HPC transdermal film, F2 in raw ACV transdermal film, F3 oleic acid used instead of GO and prepared similarly to ACV GO SNEDDS transdermal films, were all further investigated for ex vivo skin permeation and pharmacokinetic fate, and these results were compared with commercially available 5% ACV topical cream.

### 4.7. Ex Vivo Skin Permeation Study of ACV-GO SNEDDS Transdermal Films

The results of ex vivo skin permeation of ACV from F1 (optimized ACV-GO SNEDDS was loaded in HPC transdermal film), F2 (raw ACV transdermal film), F3 (oleic acid instead of GO and prepared similarly ACV GO SNEDDS transdermal films), and commercially available 5% ACV topical cream are shown in Table 3. As seen in Table 3, the cumulative amount of ACV permeating from F1 (11327 ± 977 μg/cm^2^) was markedly greater than that from F2 (4811 ± 333 μg/cm^2^), F3 (8933 ± 741 μg/cm^2^) transdermal films, and commercially available 5% ACV topical cream (7772 ± 568 μg/cm^2^). The enhancement factor for F1, which is optimized by ACV-GO-SNEDDS loaded transdermal film, was substantially greater (2.35 ± 0.4) in comparison to F2 and F3 (1.85 ± 0.3) transdermal films and commercially available 5% ACV topical cream (1.61 ± 0.4). Such research findings establish the substantial contribution of SNEDDS formulation to improving ACV-GO permeation. The outcome of ex vivo skin permeation study has shown 2.3-fold amplified permeation of ACV from optimized ACV-GO SNEDDS HPC transdermal film in comparison to raw ACV transdermal film. These study findings clearly demonstrate the influence of oil and surfactant on the ingression of constricted junctions and facilitation of the ACV transport through transcellular routes across the stratum corneum. It could be anticipated that the SNEDDS components contributed substantially in expediting the evasion of ACV through both the lipid structures of the interlamellar region and corneocytes with their keratin-enriched matrix of the skin’s transcellular routes [36,37,38].

Furthermore, there is no previous study that has investigated influence of GO in enhancing drug permeation by means of SNEDDS formulation. Therefore, to assess the contribution of GO in felicitating the permeation of ACV SNEDDS via transdermal route, an additional formulation (F3) with composition 10.4% (*w/w*) oleic acid as oil phase, 64.8% (*w/w*) surfactant mixture (Tween20^®^:Span20^®^), and 24.8% (*w/w*) co-surfactant %*w*/*w* (Propylene glycol^®^) was prepared in a similar manner as ACV-GO SNEDDS with only one difference that is substituting GO by oleic acid. Furthermore, the results of ex vivo permeation clearly indicates that GO plays significant role in enhancing ACV skin permeation via SNEDDS formulation in contrast to oleic acid constituted ACV SNEDDS.

### 4.8. In Vivo Investigation of the Optimized ACV-GO SNEDDs Transdermal Film

**Figure 5:**
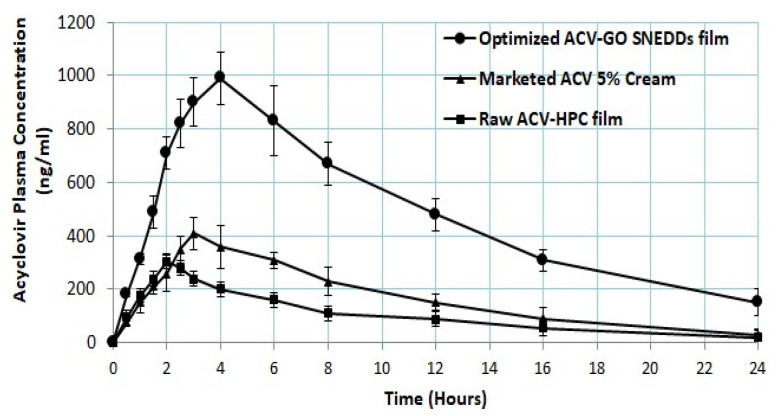
ACV plasma concentration (ng/mL) from optimized ACV-GO SNEDDS transdermal film; Raw ACV-hydroxyl propyl cellulose (HPC) film formulations and marketed cream (mean ± SD, *n* = 6).

The pharmacokinetic parameters of F2 (raw ACV HPC film), F1 (optimized ACV-GO SNEDDS transdermal film), and marketed 5% ACV cream are summarized in Table 4. It is clearly evident from Table 4 and Figure 5 that optimized ACV-GO SNEDDS transdermal film (993 ± 101 ng/mL) has significantly greater C_max_ compared to raw ACV HPC film (305 ± 42 ng/mL) and marketed 5% ACV cream (410 ± 65 ng/mL). There was a 2.2-fold increase in relative bioavailability of optimized ACV-GO SNEDDS transdermal film compared to marketed 5% ACV cream, whereas there as a 3-folds increase in relative bioavailability of optimized ACV-GO SNEDDS transdermal film compared to raw ACV-HPC film. The T_max_ value is the highest for optimized ACV-GO SNEDDS transdermal film (240 ± 30 min) in contrast to that of raw ACV HPC film (120 ± 30 min) and marketed 5% ACV cream (180 ± 30 min), which confirmed the sustained and prolonged effect of optimized ACV-GO SNEDDS transdermal film. Such steady and sustained release is desirable for alleviating cold sore conditions. The results of the in vivo study demonstrated that SNEDDS TDDS exhibited excellent potential to enhance the bioavailability of ACV.

The 2.3-fold improvement in ex vivo permeation of ACV from optimized ACV-GO SNEDDS transdermal film as compared with raw ACV HPC film corroborates the in vivo data well. The in vivo study findings revealed almost doubled C_max_ and significantly increased AUC (Table 4) of optimized ACV-GO SNEDDS transdermal film in comparison to raw ACV HPC film, whereas there was no significant difference in K_e_ for all the tested formulations.

The optimized ACV-GO-SNEDDS HPC transdermal film’s ex vivo and in vivo pharmacokinetic data demonstrated an improved and relatively greater extent of ACV absorption in contrast to raw ACV-HPC films. Such enhanced permeation and absorption is attributed to increase partitioning of ACV across the skin layers into systemic circulation. Moreover, ACV SNEDDS content, particularly oil and surfactant, exhibits tremendous potential to infiltrate the stratum corneum by improvising lipid and polar pathways for drug absorption [39]. Such improvisation in penetration pathways would allow ACV to easily cross the stratum corneum barrier, therefore resulting in increased permeation and absorption of ACV by means of SNEDDS transdermal film in contrast to raw ACV powder HPC films. Nonetheless, small globule size in the nano range would have made substantial contributions to enhancing percutaneous penetration of ACV via SNEDDS transdermal films, as an increased number of globules would become available for permeation of a definite region of the stratum corneum [40,41].

## 5. Conclusions

The optimized ACV-GO SNEDDS transdermal film demonstrated great potential for enhancing the permeation and absorption of ACV. However, the synergistic effect of GO should be explored further to establish the enhanced therapeutic activity of the combination of two antiviral agents encapsulated in the SNEDDS formulation. The present study provides strong pre-clinical evidence of enhanced pharmacokinetic parameters of developed formulation, which needs to be taken to the clinical level to come up with a valuable alternative to commercially available products with improved patient compliance for alleviating cold sores.

## Figures and Tables

**Table 1:** Observed response of formulations prepared for optimization of acyclovir (ACV)-garlic oil (GO) self-nanoemulsifying drug delivery system (SNEDDS) (Data are expressed as mean ± SD (*n* = 3)).

Formulations	A:Garlic Oil (%*w*/*w*)	B:Tween 20/Span 20 Mixture (%*w*/*w*)	C:Propylene Glycol % (*w*/*w*)	Particle Size nm ± SD
1	0.08	0.60	0.32	250 ± 5.54
2	0.08	0.66	0.26	200 ± 11.53
3	0.08	0.60	0.32	248 ± 7.12
4	0.10	0.62	0.28	192 ± 15.79
5	0.08	0.72	0.20	215 ± 12.97
6	0.16	0.62	0.22	200 ± 4.98
7	0.20	0.60	0.20	240 ± 17.43
8	0.20	0.60	0.20	239 ± 6.95
9	0.14	0.60	0.26	180 ± 10.11
10	0.14	0.66	0.20	202 ± 19.38
11	0.14	0.66	0.20	200 ± 18.11
12	0.10	0.68	0.22	177 ± 9.41
13	0.08	0.72	0.20	215 ± 12.09
14	0.12	0.64	0.24	170 ± 11.65

**Table 2:** Results showing outcomes of ANOVA test for the dependent variables (Y1).

Source	Sum of Squares	Degree of Freedom	Mean Square	F-Value	*p*-Value
Model	9095.51	8	1136.94	915.85	<0.0001
Linear Mixture	992.28	2	496.14	399.66	<0.0001
AB	917.05	1	917.05	738.72	<0.0001
AC	3318.03	1	3318.03	2672.82	<0.0001
BC	819.49	1	819.49	660.13	<0.0001
A²BC	85.02	1	85.02	68.48	0.0004
AB²C	204.01	1	204.01	164.34	<0.0001
ABC²	6.86	1	6.86	5.52	0.0655
Residual	6.21	5	1.24	-	-
Lack of Fit	1.71	1	1.71	1.52	0.2855
Pure Error	4.5	4	1.13	-	-
Cor Total	9101.71	13	-	-	-

**Table 3:** Parameters of permeation of ACV across rat skin for different formulations. Data are expressed as mean ± SD (*n* = 3).

Parameters of Permeation	F1	F2	F3	Commercial ACV (5%) Cream
Cumulative amount permeated (μg/cm^2^)	11327 ± 977	4811 ± 333	8933 ± 741	7772 ± 568
Steady state flux, Jss, (μg/cm^2^/min)	47.128 ± 6.2	17.433 ± 2.4	34.167 ± 4.9	29.308 ± 3.1
Permeability coefficient, Pc, (cm/min)	(3.9 ± 0.3) × 10^−3^	(1.5 ± 0.2) × 10^−3^	(2.9 ± 0.4) × 10^−3^	(2.1 ± 0.2) × 10^−3^
Diffusion coefficient, D, (cm^2^/min)	(12.3 ± 0.9) × 10^−3^	(4.6 ± 0.5) × 10^−3^	(8.5 ± 0.7) × 10^−3^	(5.9 ± 0.6) × 10^−3^
Relative permeation rate (RPR)	1.457 ± 0.6	0.619 ± 0.3	1.149 ± 0.5	-
Enhancement factor (EF)	2.35 ± 0.4	-	1.85 ± 0.3	1.61 ± 0.4

**Table 4:** Comparative pharmacokinetic parameters of different ACV film formulations and marketed cream (mean ± SD, *n* = 6).

PK Parameters	Raw ACV-HPC Film	Optimized ACV-GO SNEDDs Film	Marketed ACV 5% Cream
C_max_ (ng/mL)	305 ± 42	993 ± 101	410 ± 65
T_max_ (min)	120 ± 30	240 ± 30	180 ± 30
AUC_0–t_ (ng/mL h)	4213 ± 509	11,234.1 ± 1312.6	5718.3 ±811.2
AUC_0–inf_ (ng/mL h)	4566 ± 619	13,711.4 ± 1845.2	6218.4 ± 918.6
K_el_ (h^−1^)	0.133 ± 0.041	0.086 ± 0.021	0.118 ± 0.032
